# Barriers and disparities in preventive oral health care in the UK: insights from oral health professionals and consumers surveys

**DOI:** 10.1038/s41405-026-00477-2

**Published:** 2026-08-25

**Authors:** Michelle Wodke, Munisha Mangal, Alyson Axe, Billy Franks, Kate Fabrikant

**Affiliations:** 1 Medical and Scientific Affairs, Haleon, Weybridge, Surrey, UK; 2 Clinical Research & Development, Haleon, Leiden, The Netherlands

**Keywords:** Preventive dentistry, Dental epidemiology

## Abstract

**Objective/Aim:**

Preventive oral health care (POHC) is an integral aspect of overall health, yet oral health professionals (OHPs) frequently encounter challenges with patient adherence to preventive recommendations. This study aims to identify barriers to effective POHC implementation examining perspectives from both consumers and OHPs. Understanding these barriers will support targeted interventions to enhance public awareness and promote proactive oral health behaviours.

**Materials and methods:**

Two UK-wide research surveys were conducted to capture oral health habits and the integration of preventive advice during routine dental visits. The first survey targeted OHPs (*n* = 505) and consumers (*n* = 2000), exploring attitudes toward POHC and compliance. The second survey involved parents (*n* = 1004) and children (*n* = 1004) to assess parental influence on paediatric oral health practices.

**Results:**

Findings revealed notable variations in attitude towards POHC in consumers across demographics, including gender-based differences. Additionally, a misalignment between consumer expectations and OHPs perceptions was observed. Children and parent survey results further highlighted that parental awareness and attitudes substantially influenced children’s preventive behaviours and confidence, with notable regional variations.

**Conclusion:**

This research highlights the need to involve both consumers and OHPs to improve POHC for better outcomes in oral health. There is an opportunity for the oral health industry to address the identified barriers through tailored consumer-facing educational campaigns and support relevant training programmes, ensuring that all OHPs are better equipped. These efforts could build a foundation for improved quality of life and lifelong oral health in the UK.

## Introduction

Oral health continues to pose a significant public health burden in the UK, exerting pressure on dental services and resources [1] Despite recent advancements in dental care, preventive oral health care (POHC) issues remain significantly high, with persistent gaps between policy and practice [2] In 2024, the Oral Health Foundation reported that over three-quarters of adults (76%) experienced dental problems in the past year, and only 68% adhered to the recommended twice-daily brushing regime [1]. These statistics highlight the strong improvement to be made within POHC including adherence and systemic challenges. According to the UK Department of Health and Social Care, POHC comprise evidence-based measures aimed at reducing the risk and progression of oral disease through the promotion of effective oral health hygiene and modification of behavioural risk factors [3]. POHC also involves guidance on behaviours known to influence oral health outcomes, including dietary practices related to sugar intake, tobacco use and alcohol consumption. In addition, it encompasses the encouragement of regular dental attendance to enable reinforcement of preventive advice and early identification of disease [3].

Prevention is viewed as the cornerstone of dental care and it is fundamental in providing the public with long-term oral health, yet the key preventive behaviours such as regular visit to dental clinics and toothbrushing twice a day are not universally adopted [2]. There is further need to finetune preventive messaging to empower more people to engage in appropriate oral health hygiene, which can reduce the costs and risk of future invasive treatments [4–6]. Oral health professionals (OHPs) play a crucial role in shaping oral health habits and the relationship between consumer and OHPs is central to providing valuable knowledge and guidance. When discussions aren’t suitably tailored to the consumer, information may not be well-received, leading to feelings of being uninformed and less motivated, which can create barriers to effective care [2] However, many OHPs reported feeling confused or frustrated when consumers didn’t change their behaviour. As a result, preventable or manageable oral health issues often persist, leading to a perception that their efforts and time have been ineffective [7, 8].

This study aimed to examine variation in POHC in the UK using data from two nationwide surveys capturing both consumer and OHP perspectives. The analysis focused on three key dimensions of variation in POHC, including gender-related differences, regional variation, and misalignment between consumer expectations and OHP-reported practices. While gender and regional differences may indicate potential inequalities where they reflect systematic disparities in access to, delivery of, or uptake of POHC, differences between consumer and OHP perspectives are more appropriately considered indicators of service alignment, communication, and expectations.

By examining patterns in the delivery and receipt of POHC across these dimensions, this study provides insights into structural, behavioural, and perceptual factors that may influence POHC experiences and outcomes in the UK. In doing so, the study provides evidence to inform more equitable preventive service delivery, improve alignment between consumers and OHPs, and support policy and practice interventions designed to reduce disparities in access to and uptake of POHC.

## Materials and methods

### Participant recruitment

This cross-sectional online study comprised two UK-wide surveys (OHPs and consumers; parents and children) and was designed to explore a broad range of perspectives, supporting descriptive analyses rather than formal hypothesis testing. Participants were recruited through a market research consultancy, using databases sourced from a range of trusted panel partners. The questionnaires were designed in accordance with the Market Research Society code of conduct, based upon the ESOMAR principles [9, 10]. Respondents initially joined the panels through a combination of online and offline sources, completing an online profiling questionnaire which was verified through referrals, wealth registers, and IP addresses to ensure data integrity. All data was stored anonymously, allowing targeted recruitment across specific sectors and demographic groups. Surveys involved participants from 12 UK regions. Detailed regional distribution is tabulated in Tables 1–4. The study was retrospective in nature, as data were collected at a single point in time without follow-up.

**Table 1:** Oral health professional survey participants

Demographics			
	Number	Sample percentage	UK population percentage
Gender			
Male	250	49.51%	49.24%
Female	255	50.50%	50.76%
Age			
23–34	166	32.87%	15.84%
35–44	210	41.58%	13.30%
45–54	98	19.41%	12.54%
>55	31	6.14%	31.82%
Region			
East of England	45	8.91%	9.47%
Greater London	83	16.44%	13.10%
East Midlands	30	5.94%	7.31%
West Midlands	50	9.90%	8.91%
North East	27	5.35%	3.97%
North West	53	10.50%	11.13%
Northern Ireland	23	4.55%	2.81%
Scotland	36	7.13%	8.04%
South East	73	14.46%	13.88%
South West	32	6.34%	8.51%
Wales	28	5.54%	4.63%
Yorkshire and the Humber	25	4.95%	8.19%
Professional role and consumer sector			
Job title	Number		
Dentist	141		
Dental nurse	164		
Dental hygienist	109		
Dental Therapist	28		
Dental Technician	32		
Dental consultant	31		
Majority NHS	159		
Private	157		
An even split	189		

A table to display the representation of participants in the Oral Health Professional Survey in comparison to the UK population based on gender, age, region. The job title and consumer sector of the participants are also included in the table. Highlighted rows represent a ±5% difference between the sample percentage and UK Population percentage.

**Table 2:** Consumer survey participants

Characteristic	Number	Sample percentage	UK population percentage
Gender			
Male	976	48.80%	49.24%
Female	1024	51.20%	50.76%
Age			
16–24	264	13.20%	10.66%
25–34	337	16.85%	13.36%
35–44	330	16.50%	13.30%
45–54	190	9.50%	12.54%
55+	260	13.00%	31.82%
Region			
East of England	190	9.50%	9.47%
Greater London	260	13.00%	13.01%
East Midlands	148	7.40%	7.31%
West Midlands	177	8.85%	8.91%
North East	83	4.15%	3.97%
North West	223	11.15%	11.13%
Northern Ireland	55	2.75%	2.81%
Scotland	167	8.35%	8.04%
South East	277	13.85%	13.88%
South West	158	7.90%	8.51%
Wales	92	4.60%	4.63%
Yorkshire and the Humber	170	8.50%	8.19%
Characteristic		Number	
Patient Sector			
NHS Patient		1231	
Private Patient		346	
Mix of both		171	
Professionals		157	
Prefer not to say		95	

A table to display the representation of participants in the Consumer Survey in comparison to the UK population based on gender, age, region. The consumer’s patient or professional category is also included in the table. The term ‘consumers’ refers to individuals who reported to utilise healthcare services. Highlighted rows represent a ± 5% difference between the sample percentage and UK Population percentage.

**Table 3:** Children survey participants

Characteristic	Number	Sample percentage	UK population percentage	Adjusted UK population percentage
Gender				
Male	543	54.08%	49.24%	
Female	461	45.92%	50.76%	
Age				
4	53	5.28%	1.10%	11.85%
5	118	11.75%	1.11%	11.96%
6	132	13.15%	1.14%	12.28%
7	163	16.24%	1.17%	12.61%
8	166	16.53%	1.16%	12.50%
9	164	16.33%	1.17%	12.61%
10	137	13.65%	1.20%	12.93%
11	71	7.07%	1.23%	13.25%
Region				
East of England	94	9.36%	9.47%	
Greater London	122	12.15%	13.11%	
East Midlands	87	8.67%	7.31%	
West Midlands	101	10.06%	8.91%	
North East	45	4.48%	3.97%	
North West	92	9.16%	11.14%	
Northern Ireland	13	1.30%	2.81%	
Scotland	85	8.47%	8.04%	
South East	150	14.94%	13.89%	
South West	71	7.07%	8.51%	
Wales	40	3.98%	4.63%	
Yorkshire and the Humber	104	10.36%	8.19%	

A table to display the representation of participants in the Children Survey in comparison to the UK population based on gender, age, region. The adjusted percentages were calculated in the age groups to normalise the group percentages based on the UK population distribution, allowing for direct comparison. Highlighted rows represent a ±5% difference between the sample percentage and UK Population percentage.

**Table 4:** Parent survey participants

Characteristic	Number	Sample percentage	UK population percentage	Adjusted UK population percentage
Gender				
Male	262	26.10%	49.24%	
Female	742	73.90%	50.76%	
Age				
19–24	1	0.10%	7.17%	9.17%
25–34	278	27.70%	13.36%	17.09%
35–44	566	56.38%	13.30%	17.01%
45–54	155	15.44%	12.54%	16.04%
55+	4	0.340%	31.82%	40.70%
Region				
East of England	94	9.36%	9.47%	
Greater London	122	12.15%	13.10%	
East Midlands	87	8.67%	7.31%	
West Midlands	101	10.06%	8.91%	
North East	45	4.482%	3.97%	
North West	92	9.16%	11.13%	
Northern Ireland	13	1.30%	2.81%	
Scotland	85	8.47%	8.04%	
South East	150	14.94%	13.88%	
South West	71	7.07%	8.51%	
Wales	40	3.98%	4.63%	
Yorkshire and the Humber	104	10.36%	8.19%	

A table to display the representation of participants in the Parent Survey in comparison to the UK population based on gender, age, region. The adjusted percentages were calculated in the age groups to normalise the group percentages based on the UK population distribution, allowing for comparison. Highlighted rows represent a ±5% difference between the sample percentage and UK Population percentage.

#### Participant characteristics

All participant characteristics with comparisons to UK sample percentages are summarised in Tables 1–4 [11].

#### OHP and consumer surveys

Two surveys were conducted to collect data from OHPs and consumers in the UK. The first survey incorporated 505 OHPs, across various job titles including dentist, dental nurse, dental hygienist, dental therapist, dental technician and consultant. Among these, 250 OHPs were males, and 255 females, with age groups ranging from 23 to 55 years and above. They also reported the predominant consumer sector they treated, categorised as majority NHS, majority private, or an even split of NHS and private (Table 1).

The consumer survey involved 2000 nationally representative UK consumers, including 976 males and 1024 females, with age groups ranging from 16 to 55 years and above. Participants were grouped according to their consumer characteristics: NHS, Private, mixed (Private and NHS), or professionals. In the context of the survey, the term ‘consumers’ referred to individuals who reported to utilise healthcare services (Table 2). For the OHPs, questions gathered insights into consumer demographics, awareness and use of preventive care guidelines, and their practices and attitudes towards offering preventive care (Supplementary Information 1). For consumers, questions aimed to gather information about the participants’ dental care practices, their experiences of POHC, and their preferences for receiving advice (Supplementary Information 2).

#### Children and parent survey

For the children and parent survey, a total of 2008 participants were recruited, including 1004 parents and 1004 children. The children survey involved 543 boys and 461 girls, aged 4–11 years old, giving direct responses to questions (Table 3). Children aged 4–11 years were included to focus on early childhood, a critical period for habit formation and parental influence on oral health behaviours. While older children and adolescents represent an important group, they were excluded to maintain focus on early-life preventive behaviours and parental influence. This approach aligns with the overarching aim of public health programmes such as *Designed to Smile* [12], which focus on initiatives including fluoride-based interventions with a broader objective to promote preventive oral health behaviours in younger children. The children were not sampled along their own parents to avoid bias. Parents were defined as those who listed ‘Child(ren)’ as one of their family members and reported that at least one of their children was aged 5–11 years old in the profiling questionnaire. Children were defined as respondents aged 4–11 years who completed the survey.

The responses provided by parents reflect their views on behalf of the children in their care. The parent survey comprised 262 males and 742 females, with age groups ranging from 19-55 years and above (Table 4). The survey questions explored parents’ and children’s views on dental health, confidence, societal influences, and dental care habits (Supplementary Information 3).

### Procedure

An online quantitative methodology was used to collect the data for the surveys, and respondents were invited to participate via email. Geo-IP verification and cookie checks were implemented to ensure that respondents participated from the right country, and that the same respondent did not complete the research more than once. Survey respondents went through a second validation process that ensured the correct audience was communicated with. At this stage, respondents were asked non-leading screening questions specific to the survey they took.

### Analysis

Data collection and descriptive statistical analysis (summarised the demographic characteristics and response distribution) was conducted by a market research consultancy and tabulated by question number using Microsoft Excel. Research teams discussed and combined key points to establish themes.

Additional statistical analysis was performed using R studio (version 4.2.1) [13] *P*-values less than 0.05 were considered as statistically significant. As this study was not intended for confirmatory hypothesis testing, no adjustments were made for multiplicity of comparisons. Unpaired chi-squared (*χ*²) tests were used to examine categorical variables. A linear regression was used to assess the relationship between the percentage of a region’s OHPs reporting to be more likely to give advice to private consumers and the percentage of a region’s OHPs reporting to treat a majority (over 60%) of private consumers (*p* ≤ 0.05). Direct regression on individual OHPs was not possible as, by design, only aggregate OHP data were collected from the survey.

### Ethical approval

Participants were recruited via Censuswide using verified panel partners, and all data were collected anonymously. No interventions or changes to care were involved, and individual consent was not required. Health Research Authority (HRA) decision tool [33] was used for determining the type of study. Based on the tool’s guidance, the study was identified and classified as research.

## Results

Three themes were established across all the results: Gender-specific differences, the misalignment of expectations, and regional differences.

### Gender-specific differences

From the children’s direct responses, there were consistent findings across both genders. Most children reported liking their teeth (boys: 86%, girls: 89%) and moderate levels of teeth embarrassment (boys: 35%, girls: 37%). Boys reported slightly less negativity regarding their oral health compared to girls (62% vs 57%), yet most children self-reported to brush their teeth twice a day (85% vs 84%), with no significant gender differences.

**Fig. 1:**
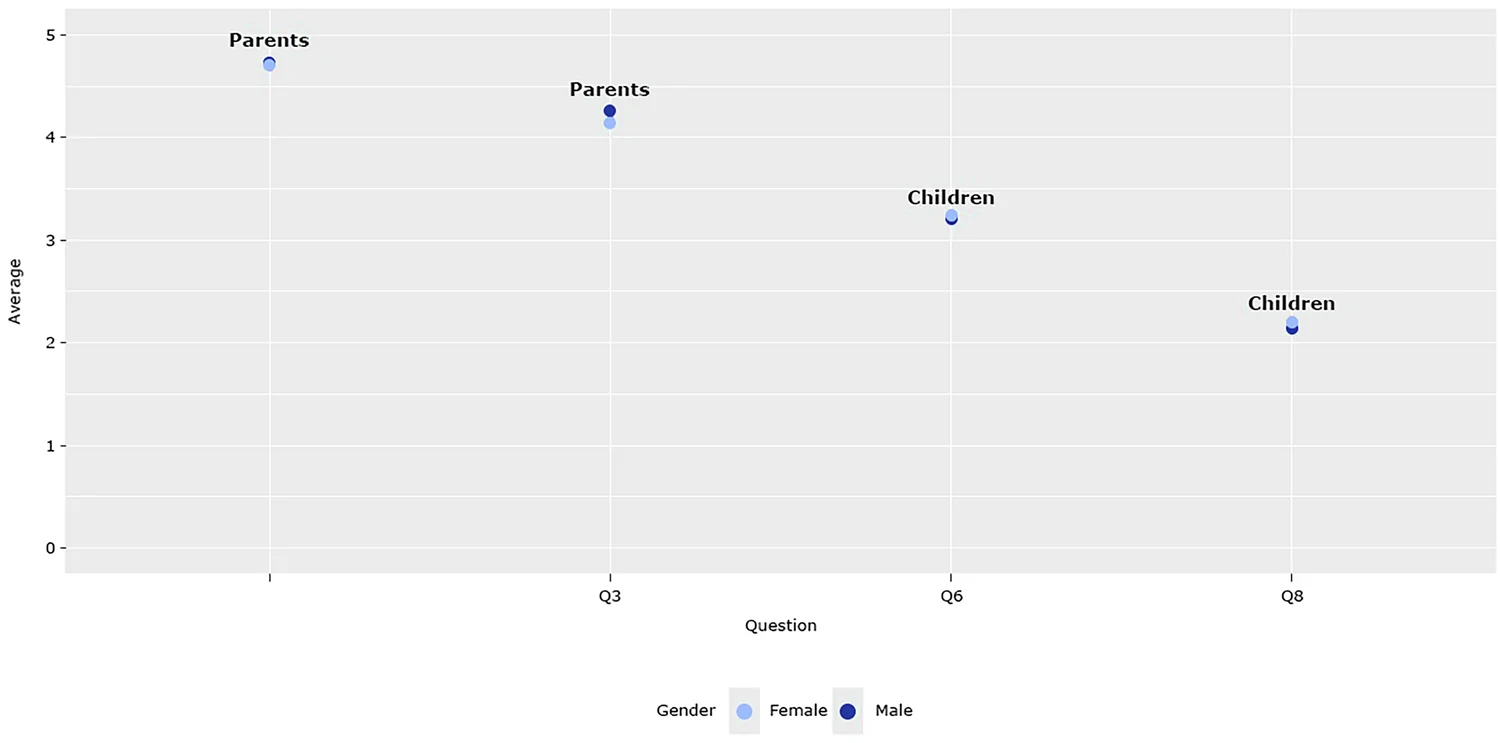
A plot to show gender-specific differences in parents and children. This plot illustrates gender-specific differences in average response rankings (on a scale of 2–5) to selected survey questions from parents and children, refer to Supplementary Information 3 for all questions. Parent responses are shown for: Q1: How important or unimportant do you believe dental health is for a child’s confidence and self-esteem? Q3: How confident or unconfident are you that you know how to keep your child’s teeth healthy? Children’s responses are shown for: Q6: Do you like your smile/how your teeth look? Q8: Have you ever been embarrassed to smile or laugh due to how you felt about your teeth? The children were not sampled along their own parents and their responses were provided by their parents. The parents in this sample were defined as those who listed ‘Child(ren)’ as one of their family members and reported that at least one of their children was aged 5–11 years old in the profiling questionnaire.

Regarding the parent results, fathers reported slightly more awareness of society’s obsession with ‘perfect teeth’ in their children (48% vs 41%) and more frequent noticing of signs of self-consciousness or low confidence in their children compared to mothers (10% vs 7%). However, there were no significant differences observed between parent genders in the awareness of preventive care’s importance for their child’s overall confidence and self-esteem (*p* > 0.05). Figure 1 displays the parent and children’s responses for both genders.

**Fig. 2:**
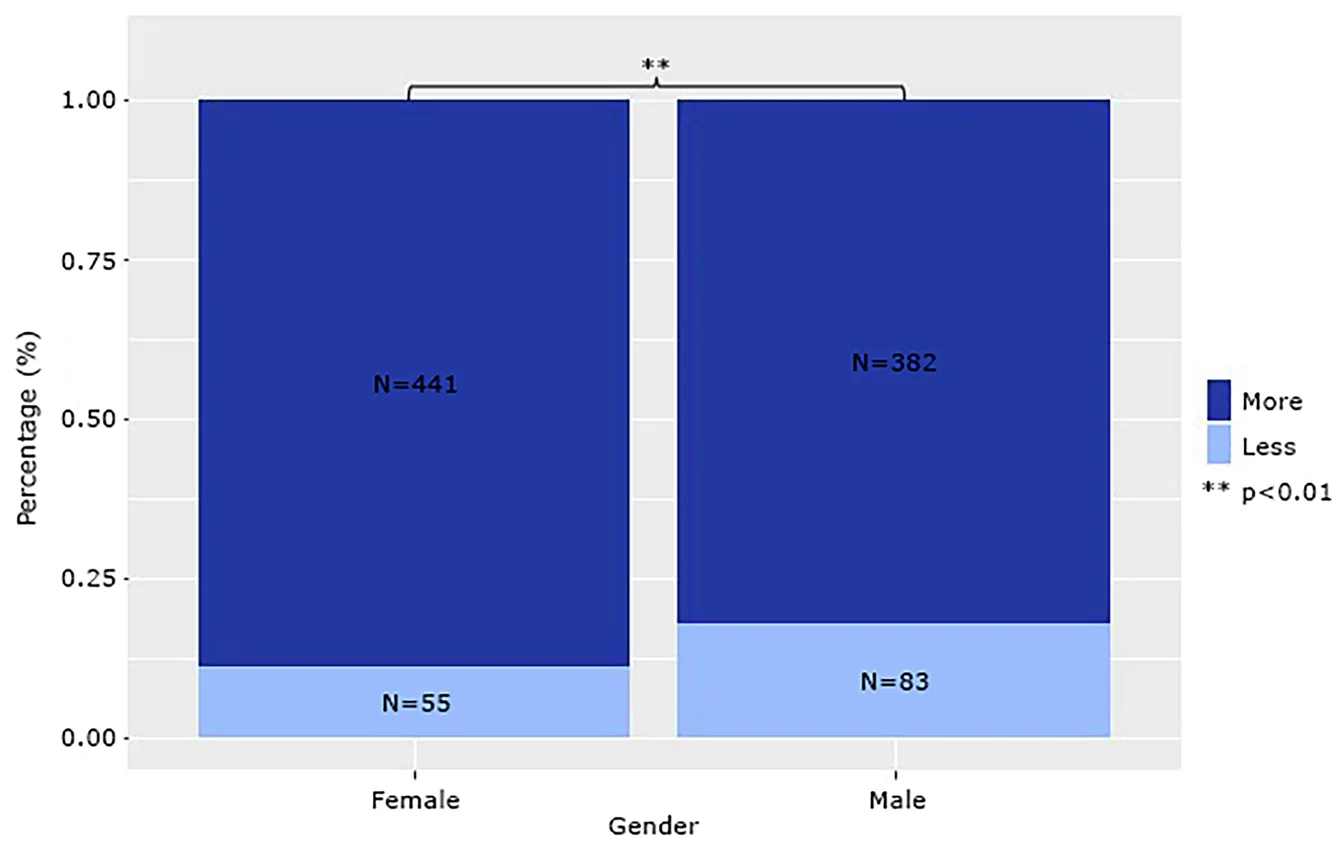
A bar graph to show the percentage of female and male consumers wanting to know more (net) or less (net) about preventive oral healthcare. Data used in this bar graph is taken from female and male responses to Question 15 in the consumer survey: Do you want to learn more, or less, about preventive care advice on your oral health? Refer to Supplementary Information 2 for all survey questions. An unpaired chi-squared test was conducted and found statistically significant difference between genders (*X*^2^ (1) = 8.38, *p* = 0.0028). The double asterisks (**) indicate a statistically significant difference of *p* < 0.01. Where N number of female and male consumers wanting to know more (net) or less (net) about preventive oral healthcare

**Fig. 3:**
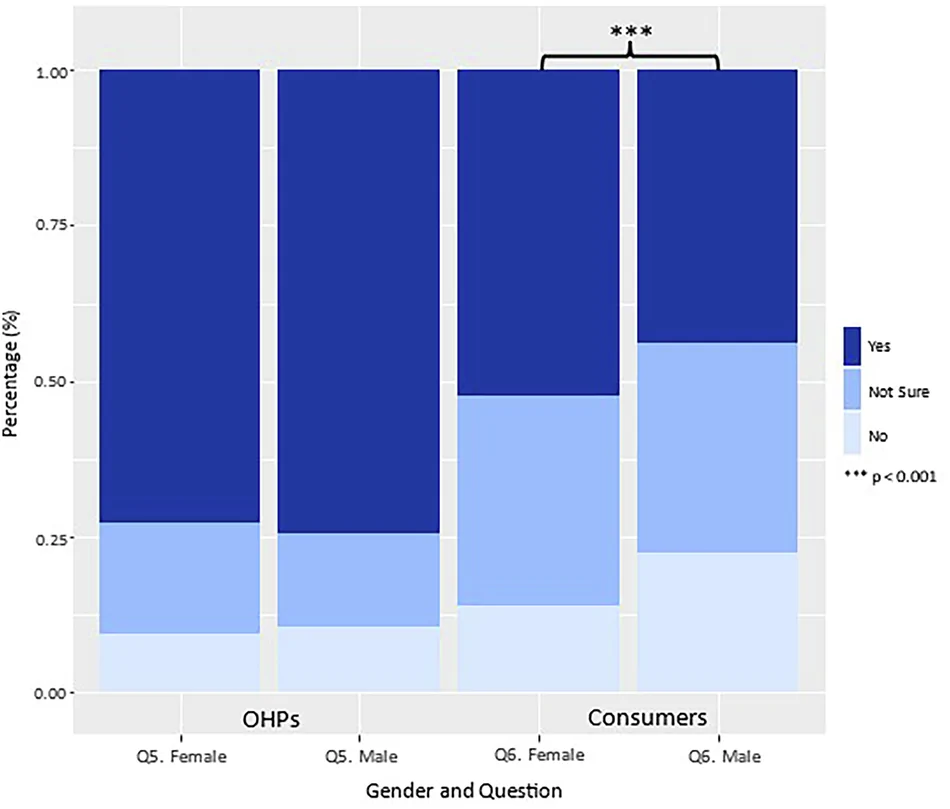
A bar chart of survey responses to NHS preventive care awareness by gender and question in OHPs and consumers. Data used in this bar graph is taken from female and male responses to Question 5 from the OHPs Survey and Q6 in the consumer survey (To the best of your knowledge, is preventive oral healthcare advice offered on the NHS?), refer to Supplementary Information 1 and 2 for all survey questions. An unpaired chi-squared test was conducted to determine statistical significance. The triple asterisks (***) indicate a statistically significant difference between genders (*X*^2^ (2) = 27.8, *p* = 9.12 × 10^−7^). There was no statistically significant difference found between genders in OHPs (*X*^2^ (2) = 0.62, *p* = 0.73).

However, an unpaired Chi-Squared test found statistically significant differences between genders in consumers’ responses to wanting to learn about preventive care advice (*X*^2^ (1) = 8.92, *p* = 0.0028), as shown in Fig. 2. Significant differences were also found between genders in consumers’ knowledge of NHS offering preventive care advice (*X*^2^ (2) = 27.8, *p* < 0.001), yet no significant differences were found in OHP results of NHS awareness (*X*^2^ (2) = 0.62, *p* = 0.73), shown in Fig. 3.

Moreover, there were consistent responses between genders in OHPs’ awareness of the Delivering Better Oral Health Toolkit (males: 77%, females: 76%) and the perceived benefits of preventive action on OH (males: 85%, females: 88%).

### The misalignment of preventive care expectation between consumers and OHPs

A misaligned view of preventive advice was clearly observed across all results. Among consumers, only 48% reported to have knowledge of NHS offering POHC advice and over a third (34%) were uncertain. Additionally, only three quarters (75%) of OHPs working with the NHS report their knowledge of preventive care advice being part of the current NHS contract.

While 73% of consumers and 65% of OHPs believed that all check-ups should include POHC advice, only 47% of consumers reported actually receiving it and just 34% of OHPs reported they consistently provide it. Additionally, 61% of dentists believed that all or most consumers act on their preventive advice, yet 74% of consumers reported to act on preventive advice given by their dentist. Survey results also found that close to a third of OHPs (30%) do not offer POHC advice as consumers simply ‘don’t ask for it’, and 44% of OHPs reported that ‘consumers asking for it more frequently’ would enable them to offer it more.

### Regional differences: NHS vs private care

**Fig. 4:**
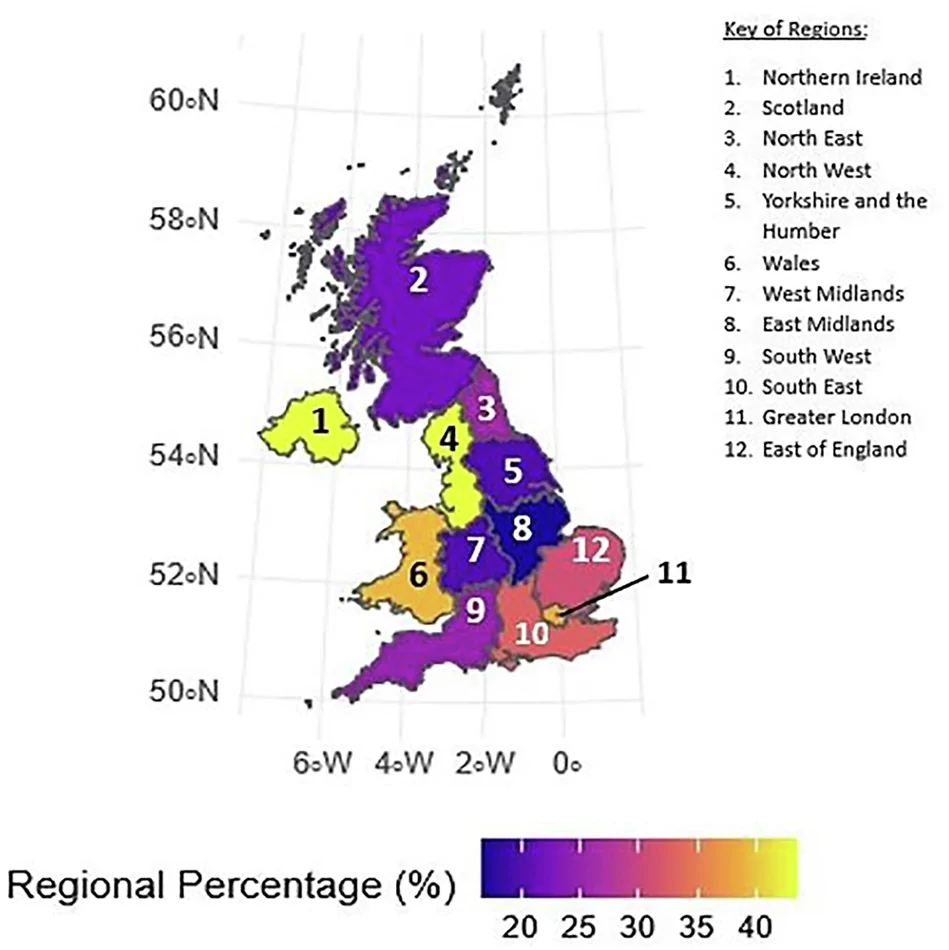
A UK regional heat map of the percentages of OHPs reporting to see a majority of private consumers in their practice. Data used in this heat map is taken from Question 2 in the OHP survey (What best defines the split of patients you see in your practice?), refer to Supplementary Information 1 for all survey questions. The regional percentage colour key refers to the percentage of OHPs reporting to treat a majority (over 60%) of private consumers in each specific region in the United Kingdom.

The regional differences of OHPs reporting to treat certain consumer sectors are visualised in Fig. 4. The regions with the highest percentages of OHPs treating most NHS consumers were Northern Ireland (NI) and the Northwest, while the lowest percentages were observed in the East Midlands, West Midlands, Scotland, and Yorkshire and the Humber.

OHPs’ awareness of the NHS offering POHC advice was lowest in Wales (43%) and Scotland (56%), but highest in the Northwest (87%). In Wales, 50% of OHPs reported seeing an even split of NHS and private consumers, yet only 32% often offered POHC advice. Furthermore, the highest percentage of OHPs (92%) agreed upon the importance of preventive care in Yorkshire and the Humber, contrasting with NI (48%) and Wales (54%).

**Fig. 5:**
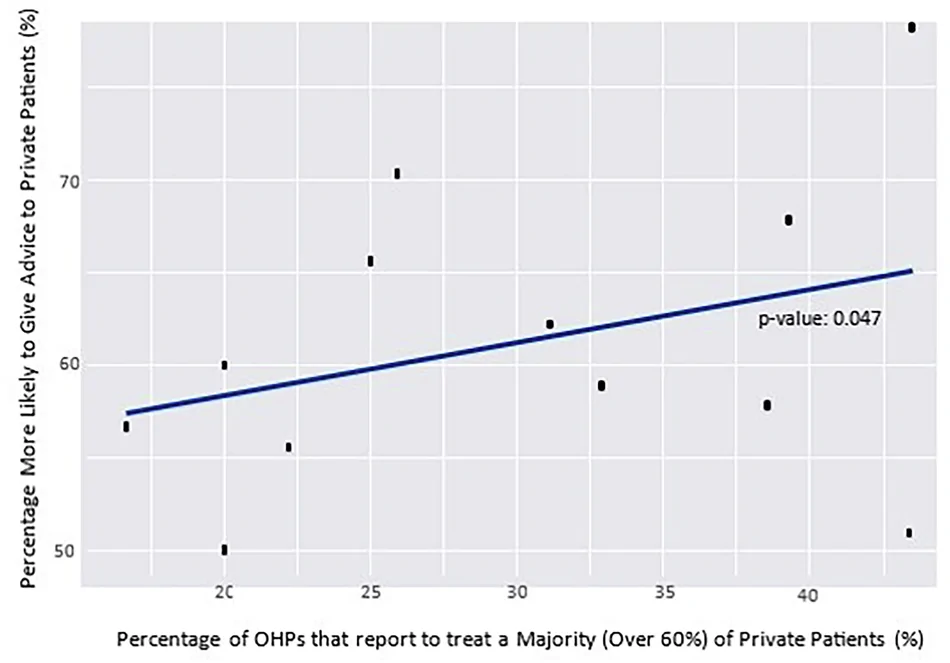
A scatter plot to show the correlation between OHPs that report to be more likely to give advice to private consumers and those who report to treat a majority (over 60%) of private consumers. Data used in this scatter plot is taken from Question 15 (*y*) Question 2 (*x*) in the OHP survey, refer to Supplementary Information 1 for all survey questions. A linear regression was fit to assess statistical significance between the two data sets and reported a significant association between OHPs reporting to be more likely to give advice to private consumers, and those reporting to treat a majority of private consumers (*R*^2^ = 0.085, coefficient = 0.2861, *p* = 0.047).

Furthermore, a statistically significant association was found between the percentage of dentists in a region who reported being more likely to give advice to private consumers, and the percentage who reported treating a majority (over 60%) of private consumers (*R*^2^ = 0.085, coefficient = 0.2861, *p* = 0.047), as illustrated in Fig. 5.

Consistent results in the consumer survey were found between NHS and private consumers’ attitudes toward POHC. Most consumers reported receiving advice verbally from their dentist (NHS: 45% vs Private: 57%), finding it helpful (NHS: 88% vs Private: 93%), and acting on the advice (NHS: 73%, Private: 74%). Similar responses were seen when asked if their dentist provided advice without them asking (NHS: 82%, Private: 83%).

In the children and parent survey results, Scotland (93%) and East of England (90%) had the highest reported oral health satisfaction rates among children, while NI (77%) and Wales (75%) had the lowest. Only 50% of children in Wales reported to visit the dentist for regular check-ups, compared to 76% in the Northeast. Additionally, NI had the highest percentage of parents reporting uncertainty about psychological issues due to oral health (38%), but only 54% reported to believe OH’s importance for a child’s overall confidence and self-esteem, compared to 84% in Scotland.

## Discussion

The findings of this study reveal critical gaps and disparities in the delivery and perception of POHC across the UK, highlighting misaligned expectations between consumers and OHPs, gender-specific differences in attitudes, and regional variations in service provision. These insights highlight the urgent need for tailored strategies that address systemic, educational, and behavioural barriers to improve oral health outcomes.

Gender-specific differences indicate that preventive health engagement is influenced by behavioural and perceptual factors, with female consumers demonstrating more proactive health-seeking behaviour. Our findings support previous studies indicating that female participants reported greater interest in learning about POHC and higher awareness of its availability through the NHS compared to males [14, 15]. No significant differences in the survey results of parents and children suggest that female consumers’ higher motivation to learn about preventive oral health may reflect differences in awareness or engagement, although casual relationships cannot be established. The absence of similar findings in parents and children suggests that these differences may not be consistent across all population groups. By recognising this, preventive oral health initiatives can be informed to customise programmes and balance health behaviours across genders, ensuring programmes are appealing and relevant to all [16, 17].

On the other hand, there were consistent responses across genders in the OHP results, showcasing a uniformity which may suggest a degree of consistency. However, this study did not assess training directly, and this interpretation should be made with caution [2, 18]. While broader policy developments may influence practice, these were not assessed in this study [19]. This research reveals gender-specific consumer motivations in oral health, suggesting that OHPs might need to consider tailoring their communication accordingly. A structured framework for preventive advice may help ensure consistency, while allowing flexibility in how advice is communicated to meet individual patient needs.

The findings also demonstrated a misalignment of expectations in POHC, suggesting systemic inefficiencies in communication and implementation. This misalignment likely reflects unclear care pathways and inconsistent integration of preventive guidance within routine practice. Creating clearer care pathways and well-defined strategies could help align expectations with actual services, as previous research has suggested a need for better communication and education to bridge this gap [20–24].

Additionally, past studies have shown that consumer’s lack of acceptance, interest, and compliance, or general confusion around prevention programmes, appeared to be a strong barrier to their oral heath action [20, 25]. Therefore, establishing prevention as a shared goal through a consumer-centred approach and strong communication is crucial for achieving exceptional care [2, 21, 25]. These findings highlight the need to increase health literacy levels in consumers to empower productive interaction with care teams, and ensure that dental teams are maximising their communications, which will support more effective preventive care and advice.

Furthermore, a misalignment was also observed in parental views. Improved education and communication are needed to align parental perceptions with actual needs, ensuring children’s development in their understanding of oral health is being addressed. While primary school education provides a foundation, these findings reinforce the role of parents as primary behavioural gatekeepers, indicating that interventions targeting parental health literacy could have a sustained intergenerational impact [23, 24, 26].

Differences between NHS and private care were observed in some OHP-reported practices. However, consumer-reported experiences were broadly similar across several measures, suggesting modest differences. Few studies have highlighted the key barriers commonly faced by NHS dentists, such as time constraints, financial pressures, and high consumer volumes, which hinder their ability to provide comprehensive care, as private OHPs do not report to encounter these as often [2, 26]. By addressing these barriers through structural reforms of oral healthcare provision, the preventive care could be enhanced, aligning it more closely with the standards observed in private practices.

Regional differences in oral health satisfaction reflect underlying inequalities in access, funding, and programme implementation, within a system where dental services and public health programmes are devolved across the UK, resulting in distinct policy frameworks, funding structures, and service delivery models. These structural differences may contribute to the variation observed across regions [27, 28].

Moreover, Wales’ ‘Designed to Smile’ programme and NI’s ‘Happy Smiles’ programme have shown variable uptake and reported outcomes due to access and real-term funding constraints and implementation challenges, alongside socioeconomic disparities and insufficient public health education [12]. Both programmes faced funding challenges in maintaining and expanding their reach within existing resource limits. As of 2020, 67.3% of children and 51.4% of adults accessed NHS dental services in Wales, with similar figures in NI [12, 29]. While some outcomes, such as reductions in general anaesthetic rates in NI have been reported, the number of children requiring general anaesthetic for dental treatment decreased from 8856 in 2004 to 5172 in 2014. Oral Health Improvement plans for children and older persons [29, 30].

In contrast, Scotland showed high satisfaction rates, regular dental check-ups, and parental awareness, likely due to the removal of financial barriers and the 2018 oral health improvement plan [30]. Therefore, despite being reported as a mainly NHS-consumer-treated region, the comprehensive, well-funded and well-implemented public health initiatives have led to better oral health outcomes and higher satisfaction rates being reported in Scotland.

These regional differences underscore the need for targeted public health initiatives and systemic reforms to ensure equitable access to oral healthcare across the UK. Addressing these issues can help bridge the gap between NHS and private services, ensuring more consistent and effective preventive care for all.

While our study provides valuable insights into the barriers to effective POHC in the UK, several limitations must be considered. Self-reported data introduces potential bias, which can affect the validity of our findings. Additionally, as a cross-sectional study, our results only offer a snapshot in time, limiting the ability to track changes longitudinally and generalise to other cultures. Moreover, the samples were not fully representative of UK age groups, which may also affect the generalisability of our findings, as the views and experiences of these age groups might not accurately reflect those of the broader UK population. Therefore, the results should be interpreted as exploratory and hypothesis-generating rather than nationally representative. While the study sample exhibited demographic imbalances, including the underrepresentation of older adults and a predominance of female respondents among parents, these findings should be interpreted in the context of existing evidence demonstrating that age and sex can substantially influence oral health experiences, perceptions, and healthcare utilisation. Older adults often experience a greater burden of oral disease and face unique barriers to accessing oral healthcare, which may lead to perspectives that differ from those of younger or middle-aged populations [31] Furthermore, previous methodological research has shown that age- and sex-imbalanced sampling may influence survey outcomes and affect the representativeness of findings [32] Therefore, the current findings may primarily reflect the views of middle-aged and female respondents and should be interpreted cautiously when extrapolating to broader parent, consumer, and OHP populations. The findings should be interpreted with caution as the analyses were primarily descriptive and potential confounders such as gender, regional variation, and socio-economic status of the consumer base were not adjusted for in the analysis. While the analysis demonstrated a statistically significant relationship between dentists’ likelihood of advising private consumers and the proportion treating a majority of private consumers, the low R² value reflects limited explanatory power, indicating that the findings should be interpreted cautiously. The absence of these adjustments may influence observed associations and regional comparison. Future research using adequately powered multivariable approaches (e.g., logistic regression) would enable more robust evaluation of these relationships. Together, these considerations emphasise the need for ongoing, context-sensitive research to enhance POHC initiatives and guarantee tailored communication strategies to ensure OHPs feel supported and heard.

## Conclusion

This research underscores the multifaceted barriers to effective POHC in the UK. It highlights variations and misalignments, including differences in consumer awareness, professional practices, and regional delivery. While gender-related differences were observed in consumers, these were not consistent across all groups and should be interpreted cautiously. The findings suggest opportunities to improve communication, enhance health literacy, and better align preventive advice with consumer expectations. Broader system-level reforms and targeted interventions may be beneficial but require further investigation. Additionally, there is an opportunity for the oral health industry to address some of these barriers through tailored consumer-facing educational campaigns and support of relevant professional training programmes, ensuring that all OHPs are appropriately supported, helping to prevent the experienced challenges when encountering preventable oral disease. While this study focused on younger children, future research should extend to adolescents, particularly given their increasing autonomy and its potential influence on preventive care behaviours. Expanding the evidence base in this way would allow for a more comprehensive understanding of oral health behaviours across developmental stages and support the design of more effective and inclusive preventive strategies.

## Supplementary information

Oral Health Professional Survey Questions

Consumer Survey Questions

Children & Parent Survey Questions
