# Supplementary material for: Barriers and disparities in preventive oral health care in the UK: insights from oral health professionals and consumers surveys
Source: BDJ Open. 2026 Aug 25;12:86. doi: 10.1038/s41405-026-00477-2 (PMC13507190; doi:10.1038/s41405-026-00477-2)
Supplement: Supplementary file 1 [file 41405_2026_477_MOESM1_ESM.pdf]

Oral Health Professional Survey Questions

Q1. On average How many patients do you see per working day?

Up to 5

6 to 10

11 to 15

16 to 20

Over 20

Q2. What best defines the split of patients you see in your practice?

Majority NHS (over 60%)

Majority Private Patients (over 60%)

An even split of NHS and private patients

Q3. Are you aware of the Department of Health's Delivering Better Oral Health toolkit that was updated in 2021?

Yes

No

Don't Know

Q4. When talking to patients, what, if any, elements do you/would you consider to be preventative advice? Tick all that apply

Advice on additional oral hygiene products (e.g. mouth wash, floss, toothpaste)

Advice on brushing techniques

Advice on lifestyle (e.g. smoking, exercise) and its impact on oral health

Defining individual patient oral health need, agreeing next steps/tools and enabling the patient to attain their goals

Advice on caring for teeth (e.g. trauma prevention and bad habit continuation)

Advice on diet and its impact on oral health

Advice on caring for periodontal tissues

Other

I don't provide any preventative advice

Q5. To the best of your knowledge, is preventative oral healthcare advice offered on the NHS?

Yes

No

Not sure

Q6. How beneficial, if at all, do you think taking preventative action on oral care could be for your patients?

Very beneficial

Somewhat beneficial

Not very beneficial

Not beneficial at all

Not Sure

Q7. How often, if ever, do you offer preventative care advice to your patients?

Always

Often

Sometimes

Rarely

Never

Q8. How, if ever, does your practice give preventative care advice? (Tick all that apply)*

Verbally in an appointment

Through sharing information leaflets offering preventative advice

Directing patients to the NHS Website

Directing patients to an oral healthcare manufacturers' website

Through follow up correspondence (e.g. emails and texts)

Showing short videos offering preventative advice in the waiting area

Other

Q9. Why don`t you always offer preventative care advice to patients? Tick all that apply**

Appointments are too time constrained

Patients don't routinely ask for it

Budget pressures

Not part of a routine appointment

Don't have access to easily available information to give to patients (e.g. leaflets, videos, website links etc.)

Other reason

This is not part of my scope of professional work

Not available on the NHS

Q10. What, if anything, would enable you to offer preventative care advice to more patients? Tick all that apply

Information that is easily available to share with patients (e.g. leaflets, videos, website links etc.)

Patients asking for it more frequently

Longer appointment times

Making it better reimbursed under NHS

Something else

N/A nothing would make me more likely to offer preventative care advice to more patients

Q11. Which of these, if either, describe when you typically provide preventative care advice to patients? *

I proactively provide them with advice without them having to ask for it

I provide advice when patients ask for it

Neither

Q12. How would you describe the level of detail you provide your patients on this preventative care advice? *

A lot of details (e.g. full personalised action plan)

Some details (e.g. some advice and guidance)

Little details (e.g. pointing to a website)

Q13. How often, if ever, do your patients question you about preventative care advice?

All the time

Most of the time

Some of the time

Rarely

Never

Q14. How many, if any, of your patients do you think put into action the preventative care advice you offer to them? *

All patients

Most patients

Some patients

A few patients

No patients

Not sure

Q15. Are you/would you be more or less likely to offer preventative advice for private patients than NHS patients?

Much more likely

Slightly more likely

No difference

Slightly less Likely

Much less likely

I wouldn't give preventative advice to private patients

Q16. Why are you/would you be more likely to offer preventative advice for private patients than NHS patients? Tick all that apply***

The fee covers the additional time taken to offer preventative advice

Private patients are more likely to pay for further treatments if not offered preventative advice

Private patients are more engaged in sustaining their oral and systemic health

Preventative advice is seen as a premium service

Other

No particular reason

Q17. Are you/would you be more or less likely to offer preventative advice for the following people when compared to the average adult?

<u>Children and their carers</u>

Much more likely

Slightly more likely

No difference

Slightly less Likely

Much less likely

I wouldn't give preventative advice to them

<u>Elderly</u>

Much more likely

Slightly more likely

No difference

Slightly less Likely

Much less likely

I wouldn't give preventative advice to them

Disabled people

Much more likely

Slightly more likely

No difference

Slightly less Likely

Much less likely

I wouldn't give preventative advice to them

Disadvantaged backgrounds

Much more likely

Slightly more likely

No difference

Slightly less Likely

Much less likely

I wouldn't give preventative advice to them

Q18. Why are you/would you be more likely to offer preventative advice for children? Tick all that apply **

Offering preventative care young helps minimise problems further down the line

It's easier to educate children to make healthy lifestyle choices

Parents are more open to learn and implement changes

It's important to share advice on fluoride consumption

Other reason

No particular reason

Q19. To what extent do you agree or disagree with the below statements?

All oral healthcare check-ups should always offer individualised preventative care advice

Strongly agree

Somewhat agree

Neither agree nor disagree

Somewhat disagree

Strongly disagree

Not sure

Preventative care is important.

Strongly agree

Somewhat agree

Neither agree nor disagree

Somewhat disagree

Strongly disagree

Not sure

Individualised preventative oral healthcare advice should be covered by the NHS

Strongly agree

Somewhat agree

Neither agree nor disagree

Somewhat disagree

Strongly disagree

Not sure

Patients generally listen when given preventative care advice

Strongly agree

Somewhat agree

Neither agree nor disagree

Somewhat disagree

Strongly disagree

Not sure

*Respondents who ever offer preventative care advice to their patients

**Respondents who always offer preventative care advice to their patients

***Respondents who are more likely to offer preventative advice for private patients than NHS patients

****Respondents who are more likely to offer preventative advice for children and their carers
