# Supplementary material for: Barriers and disparities in preventive oral health care in the UK: insights from oral health professionals and consumers surveys
Source: BDJ Open. 2026 Aug 25;12:86. doi: 10.1038/s41405-026-00477-2 (PMC13507190; doi:10.1038/s41405-026-00477-2)
Supplement: Supplementary file 2 [file 41405_2026_477_MOESM2_ESM.pdf]

Consumer Survey Questions

Q1. When you visit a dental professional, are you an NHS or private patient?

NHS Patient

Private Patient

Mix of both

Neither, I don't visit dental professionals

Prefer not to say

Q2. How often, if at all, do you visit the dentist?*

More regularly than every 3 months

Every 3 months

Every 4-6 months

Every 7-11 months

Once a year

Once every 2 years

Once every 3 years

Less often than every 3 years

Only when I have an issue

I never visit a dentist

Not sure

Q3. How often, if at all, do you visit a hygienist?*

More regularly than every 3 months

Every 3 months

Every 4-6 months

Every 7-11 months

Once a year

Once every 2 years

Once every 3 years

Less often than every 3 years

I never visit a hygienist

Not sure

Q4. How would you describe your current lifestyle? (Tick all that apply)

Regular drinker of tea / coffee

Regularly eat a healthy diet

Regularly eat sugary drinks and food

Regular alcohol drinker (more than 14 units of alcohol a week, spread across 3 days or more)

Regular smoker

Prefer not to say

Other description

Q5. When you visit a dental professional, what, if any, elements do you consider to be preventative advice? (Tick all that apply)*

Scale and polish (the removal of all plaque and tartar from teeth)

Advice on which oral care products (toothpaste, toothbrush, floss, rinse, intradental brushes) are the best ones for you to use

Ensuring all your fillings are in good condition

Advice on how to clean between your teeth using interdental brushes or floss

Recommended maintenance plan to keep your mouth healthy

Recommendations on the best ways and frequency of brushing your teeth

Recommended course of treatment to get your mouth into a good shape

A discussion about your diet and any habits you have that may affect your mouth and overall health such as diet, smoking and drinking

No particular elements

Other elements

Q6. To the best of your knowledge, is preventative oral healthcare advice offered on the NHS?

Yes

Not sure

No

Q7. Thinking back to your most recent trip to a dental health professional, how, if at all, did you receive preventative care advice? Tick all that apply*

Verbally from my dental health professionals

I did not receive or see any advice

Leaflets in the waiting room

Posters in the waiting room

Speaking to a receptionist

Follow up digital correspondence from the practice on online resources

Videos show in the waiting room

Speaking to other patients

Other

Q8. How helpful or unhelpful, have you found this/these preventative care advice sources?**

<u>Leaflets in the waiting room</u>

Very helpful

Somewhat helpful

Neither helpful nor unhelpful

Somewhat unhelpful

Very unhelpful

<u>Videos show in the waiting room</u>

Very helpful

Somewhat helpful

Neither helpful nor unhelpful

Somewhat unhelpful

Very unhelpful

<u>Posters in the waiting room</u>

Very helpful

Somewhat helpful

Neither helpful nor unhelpful

Somewhat unhelpful

Very unhelpful

Verbally from my dental health professionals

Very helpful

Somewhat helpful

Neither helpful nor unhelpful

Somewhat unhelpful

Very unhelpful

Follow up digital correspondence from the practice on online resources

Very helpful

Somewhat helpful

Neither helpful nor unhelpful

Somewhat unhelpful

Very unhelpful

Speaking to other patients

Very helpful

Somewhat helpful

Neither helpful nor unhelpful

Somewhat unhelpful

Very unhelpful

Speaking to a receptionist

Very helpful

Somewhat helpful

Neither helpful nor unhelpful

Somewhat unhelpful

Very unhelpful

Other

Very helpful

Somewhat helpful

Neither helpful nor unhelpful

Somewhat unhelpful

Very unhelpful

Q9. Where, if at all, have you encountered preventative care advice on oral care before? (Tick all that apply)

Visiting a dental health professional

When I proactively search it on the internet (e.g. google search)

Through TV adverts

I don't receive any preventative care advice on oral care from any particular place

From a friend or family member

Prefer not to say

Social media (facebook, Instagram, tiktok, twitter)

When I visit the pharmacy

Lifestyle magazines

Other place

Q10. How helpful or unhelpful, have you found this/these preventative care advice sources?***

<u>Visiting a dental health professional</u>

Very helpful

Somewhat helpful

Neither helpful nor unhelpful

Somewhat unhelpful

Very unhelpful

<u>When I proactively search it on the internet (e.g. google search)</u>

Very helpful

Somewhat helpful

Neither helpful nor unhelpful

Somewhat unhelpful

Very unhelpful

<u>Social media (facebook, Instagram, tiktok, twitter)</u>

Very helpful

Somewhat helpful

Neither helpful nor unhelpful

Somewhat unhelpful

Very unhelpful

Through TV adverts

Very helpful

Somewhat helpful

Neither helpful nor unhelpful

Somewhat unhelpful

Very unhelpful

Lifestyle magazines

Very helpful

Somewhat helpful

Neither helpful nor unhelpful

Somewhat unhelpful

Very unhelpful

From a friend or family member

Very helpful

Somewhat helpful

Neither helpful nor unhelpful

Somewhat unhelpful

Very unhelpful

When I visit the pharmacy

Very helpful

Somewhat helpful

Neither helpful nor unhelpful

Somewhat unhelpful

Very unhelpful

Other place

Very helpful

Somewhat helpful

Neither helpful nor unhelpful

Somewhat unhelpful

Very unhelpful

Q11. How important, if at all, do you think preventative care is for oral health?

Very important

Somewhat important

Not very important

Not important at all

Not sure

Q12. You mentioned you have received preventative care advice before from your dental health professional. Did they offer this advice proactively, or did you have to request this information from them? If this happened more than once, please think of the last time this happened when answering the questions. **

They provided me with advice without me having to ask for it

I had to ask for the advice otherwise they might not have provided it

Can't remember

Neither

Q13. How would you describe the level of detail your dental health professional provided on this preventative care advice? **

Dentist

A lot of details (We made a clear action plan which I agreed to follow)

Some details (some advice and guidance)

Little detail (pointing to a website)

Can't remember

N/A; I did not receive preventative care advice from this kind of professional

Dental Nurse

A lot of details (We made a clear action plan which I agreed to follow)

Some details (some advice and guidance)

Little detail (pointing to a website)

Can't remember

N/A; I did not receive preventative care advice from this kind of professional

Hygienist

A lot of details (We made a clear action plan which I agreed to follow)

Some details (some advice and guidance)

Little detail (pointing to a website)

Can't remember

N/A; I did not receive preventative care advice from this kind of professional

Q14. How much, if at all, have you put in action this preventative care advice from your dental health professional? ****

Put it all into action

Put most of it into action

Put some of it into action

Put none of it into action

Can't remember

Q15. Do you want to learn more or less about preventative care advice on your oral health?

Much more

Somewhat more

The same as now

Somewhat less

Much less

Not sure

Q16. When seeking/if you were to seek preventative care advice for your oral health, where would you go? (Tick all that apply)

The Dentist

Dental Hygienist/Therapist

NHS Website

Dental Health Nurse

Doctor / GP

General websites

Not sure

Oral healthcare manufacturer websites

Ask Family/Friends

Pharmacist

Social media

Blogs

Nowhere

Other place

Q17. If you were to seek preventative care advice for your oral health, how would you prefer to receive this? (tick all that apply)

From a Dentist

Through the NHS Website

From a Doctor / GP

Not sure

Through leaflets / handouts at the dental practice

Through a search engine

Via Oral healthcare manufacturer websites

From a pharmacist

Ask Family/Friends

Through interactive video content at the dental practice

Through Social media

Nowhere

Other place

Q18. To what extent do you agree or disagree with the following statements?

<u>Preventative oral healthcare is important</u>

Strongly agree

Somewhat agree

Neither agree nor disagree

Somewhat disagree

Strongly disagree

<u>All check-ups should always offer preventative oral care advice</u>

Strongly agree

Somewhat agree

Neither agree nor disagree

Somewhat disagree

Strongly disagree

<u>All check-ups should always offer preventative oral care advice and action planning to achieve my health goals</u>

Strongly agree

Somewhat agree

Neither agree nor disagree

Somewhat disagree

Strongly disagree

<u>Oral Preventative care advice should be covered by the NHS</u>

Strongly agree

Somewhat agree

Neither agree nor disagree

Somewhat disagree

Strongly disagree

Parents are more likely to seek oral preventative advice for their children rather than themselves

Strongly agree

Somewhat agree

Neither agree nor disagree

Somewhat disagree

Strongly disagree

*Those who ever visit a dental professional

**Those who received each type of advice

***Those who have received preventative oral care from these sources

****Those who have received preventative oral care from visiting a dental health professional
