# Supplementary material for: Barriers and disparities in preventive oral health care in the UK: insights from oral health professionals and consumers surveys
Source: BDJ Open. 2026 Aug 25;12:86. doi: 10.1038/s41405-026-00477-2 (PMC13507190; doi:10.1038/s41405-026-00477-2)
Supplement: Supplementary file 3 [file 41405_2026_477_MOESM3_ESM.pdf]

Children & Parent Survey Questions

Parent Questions

Q1. How important or unimportant, do you believe dental health is for a child's overall confidence and self-esteem?

Very important

Important

Neither important nor unimportant

Unimportant

Very unimportant

Q2. How often, if ever, have you noticed signs of self-consciousness or lack of confidence in your child related to their smile or teeth / how their teeth look?

Frequently

Regularly

Sometimes

Rarely

Never

Q3. How confident or unconfident are you that you know how to keep your child's teeth healthy?

Very confident

Confident

Neither confident nor unconfident

Unconfident

Very unconfident

Q4. Do you think your child is impacted or influenced by societys obsession with picture perfect teeth (perfectly white & straight teeth)?

Yes, definitely

Yes, probably

No, not really

No, definitely not

Not sure

Q5. What negative impacts, if any, has your child ever experienced due to oral health problems or because of how their teeth look? (Tick all that apply)

My child has never experienced any negative impacts due to oral health problems or because of how their teeth look

Unsure

My child has been in pain/discomfort

My child seems less confident

My child avoids smiling

My child has been called names and been teased

Other negative impact

My child has been bullied

Prefer not to say

Children Questions

Q6. Do you like your smile/how your teeth look?

Yes, I love smiling and how my teeth look

Yes, a little bit/sort of

No, not really

No, not at all

Q7. What, if anything, makes you feel less confident? (Tick all that apply)

Nothing makes me feel less confident

Some of my teeth are missing

My teeth aren't white

I have some wonky teeth

Clothes I don't like

The way my teeth look

I think I have gappy teeth

My hair style

Other

I have braces on my teeth

Q8. Have you ever been embarrassed to smile or laugh due to how you felt about your teeth?

Yes, a lot

Yes, sometimes

No, not really

No, never

Q9. Which of the following, if any, has ever happened to you? (Tick all that apply)

None of these have ever happened to me

Someone told me I had smelly breath

Someone said something mean to me about my teeth

Someone at school said my teeth were funny

I have been teased about how my teeth look

Prefer not to say

Q10. How, if at all, do you currently take care of your teeth? (Tick all that apply)

Brushing teeth twice a day

Visiting the dentist for check-ups

Avoiding too many sugary snacks and drinks

Flossing regularly

I don't do anything to currently take care of my teeth

Other, please specify
